# An Intrinsic Oscillation of Gene Networks Inside Hair Follicle Stem Cells: An Additional Layer That Can Modulate Hair Stem Cell Activities

**DOI:** 10.3389/fcell.2020.595178

**Published:** 2020-12-10

**Authors:** Patrycja Daszczuk, Paula Mazurek, Tomasz D. Pieczonka, Alicja Olczak, Łukasz M. Boryń, Krzysztof Kobielak

**Affiliations:** Laboratory of Stem Cells, Development and Tissue Regeneration, Centre of New Technologies (CeNT), University of Warsaw (UW), Warsaw, Poland

**Keywords:** hair follicle stem cells (hfSCs), dermal papilla, niche, BMP signaling, WNT signaling

## Abstract

This article explores and summarizes recent progress in and the characterization of main players in the regulation and cyclic regeneration of hair follicles. The review discusses current views and discoveries on the molecular mechanisms that allow hair follicle stem cells (hfSCs) to synergistically integrate homeostasis during quiescence and activation. Discussion elaborates on a model that shows how different populations of skin stem cells coalesce intrinsic and extrinsic mechanisms, resulting in the maintenance of stemness and hair regenerative potential during an organism’s lifespan. Primarily, we focus on the question of how the intrinsic oscillation of gene networks in hfSCs sense and respond to the surrounding niche environment. The review also investigates the existence of a cell-autonomous mechanism and the reciprocal interactions between molecular signaling axes in hfSCs and niche components, which demonstrates its critical driving force in either the activation of whole mini-organ regeneration or quiescent homeostasis maintenance. These exciting novel discoveries in skin stem cells and the surrounding niche components propose a model of the intrinsic stem cell oscillator which is potentially instructive for translational regenerative medicine. Further studies, deciphering of the distribution of molecular signals coupled with the nature of their oscillation within the stem cells and niche environments, may impact the speed and efficiency of various approaches that could stimulate the development of self-renewal and cell-based therapies for hair follicle stem cell regeneration.

## Introduction

**FIGURE 1:**
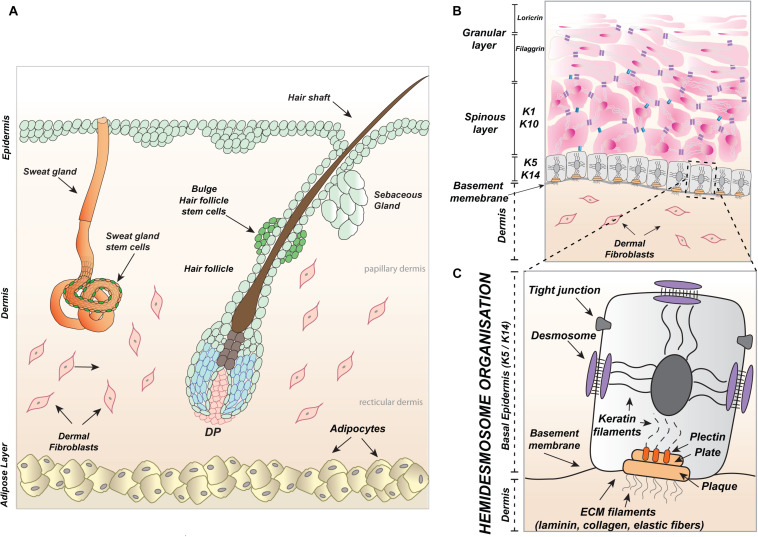
Skin and appendages. **(A)** Schematic, illustrating a section through the skin including hair follicle along with the sebaceous gland as well as the sweat gland protruding into the dermis with fibroblasts and adipocytes and underlying hypodermis. **(B)** Graphic representation of skin epidermis: basal layer with the expression of Keratin 5 (K5) and Keratin 14 (K14) above, spinous layer positive for Keratin 1 (K1) and Keratin 10 (K10) and on the top granular and cornified layers marked accordingly with Filaggrin and Loricrin. **(C)** Illustration of the junctional connection between keratinocytes of basal layer and fibroblasts of the papillary dermis, firmed by hemidesmosomes, which are composed of integrin subunits α and β attached to the basement membrane and bridged by plectin to keratin cytoskeleton filaments. The docking filaments of the basement membrane: laminin, collagen, and elastic fibers form part of the extracellular matrix (ECM) produced by keratinocytes and dermal fibroblasts. Tight adhesion of keratinocytes in the epidermis is created by tight junctions and the formation of desmosomes. The papillary dermis is primarily composed of fibroblasts, arranged in loose networks and elastic fibers of the ECM. DP, dermal papillae.

Skin is the largest organ, covering the entire human body and extending up to an area of approximately two square meters. Skin is built up of two main layers, the epidermis and the underlying dermis which are formed during embryonic development from the ectoderm and mesoderm, respectively (Figure 1A). The top part of the ectodermal skin creates the epidermis which, during morphogenesis, delves into the dermis and develops skin appendages to produce visible skin hair fibers (from hair follicles) with sebum (from sebaceous glands) and sweat glands (Figures 1A,B). Keratinocytes are the main building blocks of the epidermis and skin appendages.

They create an outside barrier with several epidermal layers (Figure 1B) and possess keratin intermediate filaments inside their cytoplasm (Figure 1C). Apart from keratinocytes, the epidermis is immersed with other cell types derived from the neural crest (melanocytes), bone marrow (Langerhans cells or dendritic cells), and epithelium (Merkel cells) (Merad et al., 2002; Mende et al., 2006; Van Keymeulen et al., 2009; Ernfors, 2010). The dermis is created by fibroblasts which form an upper and lower layer called the papillary dermis and the reticular dermis, respectively (Figure 1A). The epidermis and papillary dermis produce collagen, laminin, and elastic fibers of the extracellular matrix (ECM) which provide an anchoring interface for the basement membrane (Figures 1B,C). The part of the anchoring interface outside of the basement membrane is produced by integrin complexes (subunits α and β) present in keratinocytes in the basal or outer root sheath (ORS), known as hemidesmosomes, which connect through plectin to the cytoskeleton composed of keratin filaments (Figure 1C). Apart from fibroblasts, the additional cells localized in the skin reticular dermis are adipocytes (fat cells) (Figure 1A), immune cells like macrophages, lymphocytes, and mast cells.

**FIGURE 2:**
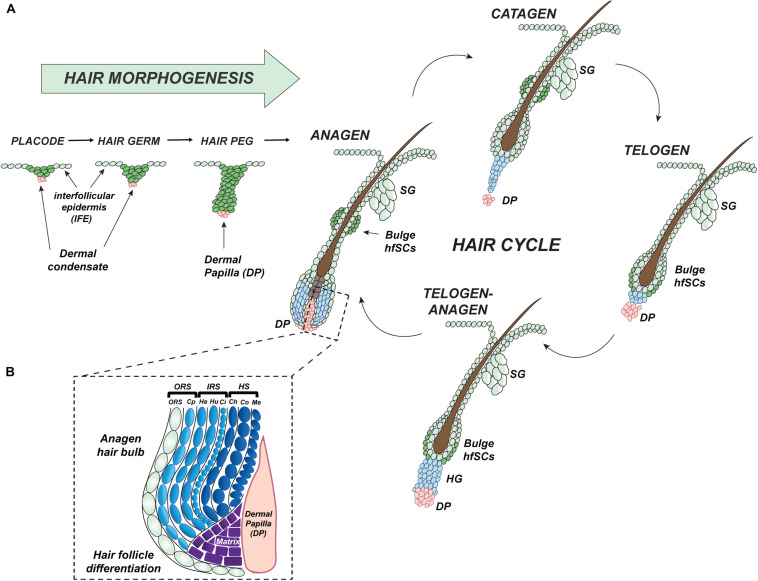
Development and cyclic regeneration of hair follicle. **(A)** Graphic stages of hair follicle (HF) morphogenesis: hair placode (initiation of HFs pattern formation), immature hair germ, hair peg, and fully developed and differentiated anagen hair by the end of first postnatal resting phase of telogen. Mature HF is built of a reservoir of hair follicle Stem Cells (hfSCs) which is connected above to an associated sebaceous gland (SG), located below the responsive hair germ (HG) with underlying dermal papilla (DP). During the cyclic hair growth, each HF undergoes phases of telogen (quiescence, resting), anagen (activation, regrowth), and catagen (degeneration). **(B)** During the growth phase, the hair bulb cells comprise actively proliferative matrix cells which undergo final differentiation creating an inner root sheath (IRS) and hair shaft (HS, protruding out of the skin surface, visible part of HF) layers of the HF mini-organ. HS, hair shaft is composed of Ch, the cuticle of the hair shaft; Co, cortex of hair shaft; Me, medulla; DP, dermal papilla; IRS, inner root sheath contains; He, Henle’s layer; Hu, Huxley’s layer; Ci, cuticle; Cp, companion layer; ORS, outer root sheath.

During early mouse embryonic development, between embryonic day 9.5 (E9.5) to 12.5 (E12.5), a single layer of the epidermis begins stratifying into the several layers of cells which create a protective wall completely impermeable before birth (Blanpain and Fuchs, 2009). Then, at E13.5, mutual interaction between secreted clues from mouse epidermis and dermis initiates the first hair follicle (HF) placode formation, which begins to delve into the dermis (Figure 2A) (Dhouailly, 1973; Li et al., 2015). Currently discovered epithelial signals released from placode, such as fibroblast growth factor 20 (FGF20) guide the initial condensation of fibroblasts to create the dermal papilla (DP), a signaling center, which is essential to complete the development of the hair follicle (Figure 2A) (Oliver and Jahoda, 1988; Millar, 2002; Huh et al., 2013).

HFs are very well organized mini-organs with extraordinary potential to undergo coordinated and spontaneous cycles of self-activated regeneration. These synchronized cycles of hair regrowth are characterized by phases of anagen (growth), catagen (degeneration), and telogen (quiescence) that are controlled by reciprocal signal interaction between hair follicle Stem Cell (hfSCs) and predominantly DP (Figure 2A) (Muller-Rover et al., 2001; Millar, 2002; Schmidt-Ullrich and Paus, 2005; Alonso and Fuchs, 2006; Rendl et al., 2008). In the growth phase of an HF’s cycle, there is sequential activation of cell proliferation: first in the highly responsive region located directly above the DP known as the hair germ (HG); second, in the hfSCs in the bulge of ORS (Figure 2A). Subsequently, cells from both structures generate a hair bulb matrix of transit-amplifying (TA) cells which differentiate into several layers of the visible hair shaft (HS) formed by the medulla, cortical layers, and the cuticle, together with the internal tunnel of the inner root sheath (IRS) (Figures 2A,B) (Greco et al., 2009). Postnatally, after morphogenesis of HFs and the epidermis is complete, two physiological independent pools of keratinocytes, one in the interfollicular epidermis (IFE) and another in the hair bulge, are responsible for different cell fates (Figure 2A) (Ito et al., 2005; Levy et al., 2005; Jaks et al., 2008; Nowak et al., 2008; Lu et al., 2012; Leung et al., 2013). During emergencies like skin injury, SCs (Stem Cells) from skin appendages, such as hair follicles, sweat glands, or nails which under normal conditions are restricted to that specific regeneration fate are capable of actively contributing to the regeneration of the epidermis (Tumbar et al., 2004; Ito et al., 2005; Levy et al., 2007; Snippert et al., 2010; Lu et al., 2012; Leung et al., 2013, 2014).

**FIGURE 3:**
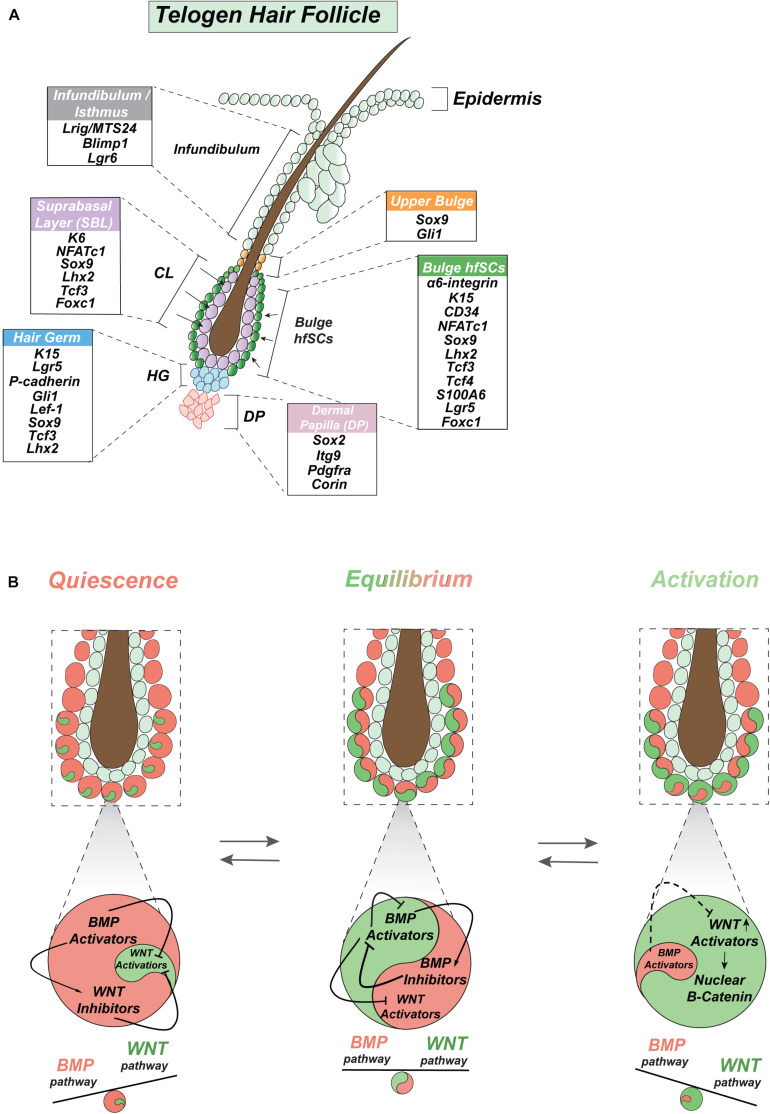
Intrinsic signaling oscillation in hair follicle stem cells. **(A)** Graphic depiction of stemness genes expressed in quiescent telogen including bulge with hfSCs along with upper bulge and infundibulum of HF, hair germ, suprabasal bulge layer (SBL) layer, and DP. **(B)** Model of proposed intrinsic oscillation in hfSCs represents intrinsic dynamic competitive oscillation along BMP signaling axis of activators and inhibitors and its reciprocal interaction between BMP and WNT pathways in SCs (stem cells) homeostasis regulation.

The sebaceous gland is located above the bulge with hfSCs and produces sebum, which forms together with HF and the pilosebaceous unit, but is independently preserved by Blimp1+ unipotent progenitor cells (Figure 3A) (Horsley et al., 2006). However, hfSCs are also capable of participating in sebaceous gland physiological restoration (Petersson et al., 2011). Similarly, Lgr6+ positive cells, localized in the ORS above the bulge in the HFs isthmus, can renew sebaceous glands as well as IFE (Figure 3A) (Snippert et al., 2010; Joost et al., 2018).

In this review, we present current views on the discoveries of molecular mechanisms that allow hfSCs to synergistically integrate homeostasis during quiescence and activation. The review also explores a model of how different populations of skin stem cells consolidate their intrinsic and extrinsic mechanisms, resulting in the maintenance of their stemness and hair regenerative potential. Primarily, we focus on the cyclic nature of HF regeneration and on the question of how the intrinsic oscillation of gene networks in hfSCs sense and influence the surrounding niche environment. We also investigate the existence of a cell-autonomous mechanism and the reciprocal interaction between key niche components and key molecular axes in hfSCs and the environment, demonstrating its critical driving force in either activation of whole mini-organ regeneration or quiescence homeostasis maintenance.

## Stemness of Hair Follicle Stem Cells and Regenerative Potential

Hair follicle stem cells are a small population of SCs residing in the outermost layer of the skin, which is referred to as the HF bulge, below the sebaceous gland (Figure 3A), at the arrector pili muscle attachment site (Cotsarelis et al., 1990; Wei et al., 1995; Fujiwara et al., 2011). The putative location of hfSCs was confirmed by pulse-chase experiments employing labeled nucleotides (Cotsarelis et al., 1990; Morris and Potten, 1999; Taylor et al., 2000) and a transgenic mouse model with doxycycline-inducible expression of a histone H2B-GFP labeling system (Tumbar et al., 2004) that proved the existence of quiescent, slow-cycling label-retaining cells (LRCs), specifically in the bulge region (Figure 3A) (Panteleyev et al., 2001). Label-retaining slow-cycling cells are already present in the early stage of HF morphogenesis and are marked by Sox9 expression (Nowak et al., 2008). This early Sox9 positive population of SCs is forming in the placode in a Sonic hedgehog (Shh) dependent way by adopting a low WNT (Wingless-type MMTV integration site family) signaling gradient as suprabasal placode cells after the asymmetric division of basal placode cells with high WNT signaling (Ouspenskaia et al., 2016). This early population of SCs is responsible for the formation of quiescence bulge SCs and delivers progeny to all skin lineages, apart from HFs sebaceous glands and IFE (Nowak et al., 2008).

Hair follicles undergo highly coordinated regenerative cycles under normal physiological conditions, with repeating phases of anagen, catagen, and telogen during a lifetime. Most of the time hfSCs are quiescent but periodically become activated to start HF regeneration (Figure 2A). In mice, the bulge as an anatomical structure becomes morphologically established during the first postnatal telogen (at P18) at the end of HF morphogenesis, then hfSCs also start to express the mature hfSC cell surface marker CD34 (Trempus et al., 2003). Together with α6-integrin, the CD34 marker can be employed to faithfully isolate basal hfSCs by fluorescence-activated cell sorting (*FACS*) (Trempus et al., 2003; Blanpain et al., 2004; Morris et al., 2004) (Figure 3A). Moreover, LRCs-hfSCs reveal the highest level of keratin K15 (K15) expression (Figure 3A) (Liu et al., 2003; Morris et al., 2004).

In the past, K15-GFP or intermittently dividing LRCs reporter mice and the application of immunofluorescence staining with α6-integrin+/CD34+ enabled isolation by *FACS* living hfSCs in the first microarray-based experiments (Blanpain et al., 2004; Morris et al., 2004; Tumbar et al., 2004). In addition to CD34, which labels more than 90% of K15-GFP+ hfSCs (Morris et al., 2004), there are high levels of other key SCs stemness markers, such as Lhx2 (LIM homeobox2), Sox9, Tcf3 (T-cell factor3), Tcf4, Lgr5 (Leu-rich repeat-containing G protein-coupled receptor5), NFATc1 (the nuclear factor of activated T-cell cytoplasmic1), and Foxc1 (forkhead box c1) (Figure 3A) (Merrill et al., 2001; Vidal et al., 2005; Nguyen et al., 2006; Rhee et al., 2006; Horsley et al., 2008; Nowak et al., 2008; Nguyen et al., 2009; Kadaja et al., 2014; Adam et al., 2015; Lay et al., 2016; Wang et al., 2016). Sox9 is crucial to maintain hfSCs stemness by Activin B/TGFβ/pSmad2 signaling that inhibits the IFE fate (Kadaja et al., 2014). Importantly, Sox9 directly regulates another stemness marker Lhx2 (Kadaja et al., 2014). The special role of Sox9 in orchestrating the formation of hfSCs has been demonstrated by its ablation with subsequent inhibition of Lhx2, Tcf3, and Tcf4 expression (Adam et al., 2015). Thus, Sox9 has been recognized as a pioneer factor coupling stemness transcription factors Lhh2, Tcf3, Tcf4, NFATc1, Tle, and Nfib along with Mediator subunit (Med1) and histone H3 acetylation on lysine 27 (H3K27ac, activation mark), which localize super-enhancers with their epicenters to maintain hfSCs (Adam et al., 2015).

In another recent study, the loss of nuclear factor IB (Nfib) and IX (Nfix) revealed the abolition of the epigenetic landscape of super-enhancers with the inability to maintain hfSCs stemness (Adam et al., 2020). In addition, expression of NFATc1 is directly controlled by canonical BMP/Smad1/5/8 signaling in the hfSCs quiescence, since the NFATc1 promoter possesses Smad’s binding sites (Horsley et al., 2008; Kandyba et al., 2013; Genander et al., 2014). BMP (Bone Morphogenetic Protein) signaling, together with Calcium/calcineurin (CN) are required to activate NFATc1, which then suppresses the cyclin dependent kinase 4 gene (Cdk4) expression, keeping the bulge in a quiescent state (Horsley et al., 2008). Another recent study discovered an additional molecular mechanism was discovered where Foxc1 activates NFATc1 and BMP signaling, as major quiescence organizers, while Foxc1 in activated bulge SCs are required to restore and preserve quiescence (Lay et al., 2016; Wang et al., 2016). Foxc1 binding sites were found in promoter or enhancer regions of genes involved in hfSCs quiescence, including Bmp2, Foxp1 (forkhead box p1), NFATc1, and Prlr. Finally, a comparison between gene expression and correlation with specific motifs for Foxc1, NFATc1, and Smad indicates cooperation of gene networks in the regulation of the quiescence state (Wang et al., 2016).

Genome-wide studies depict histone H3 tri-methylation on lysine 4 (H3K4me3) and lysine 79 (H3K79m2) as an indicator of promoters of actively transcribed genes of hfSCs, including all previously reported stemness genes, whereas differentiation genes in hfSCs are repressed by repressive H3 tri-methylation on lysine 27 (H3K27me3) (Lien et al., 2011).

One of the most important characteristics of genuine SCs is the capability to sustain their stem proliferative feature *in vitro* with a long-term self-renewing property in culture (Barrandon and Green, 1987). Indeed, hfSCs demonstrate the highest efficiency in the colony-forming assay when compared to all tested populations of keratinocytes isolated from different parts of the skin *in vitro* (Rochat et al., 1994; Oshima et al., 2001; Trempus et al., 2003; Blanpain et al., 2004; Morris et al., 2004). However, an even more significant test of SCs is their ability to maintain regenerative characteristics *in vivo* by performing a well-established assay of the chamber graft reconstitution (Lichti et al., 1993; Weinberg et al., 1993; Lichti et al., 2008). In this assay, different populations of keratinocytes are tested for SC potential by combining them with DP-enriched fraction of fibroblasts from neonate mice and then the resulting cell suspension is grafted onto the backs of immunocompromised mice to regenerate a full-thickness epidermis with appendages. As expected, hfSCs are capable of regenerating HFs, the epidermis, and the sebaceous glands when grafted into the chamber on athymic (NUDE) mice thereby demonstrating their multipotency (Blanpain et al., 2004; Morris et al., 2004; Claudinot et al., 2005). Similarly, human bulge hfSCs have been also labeled by K15 expression (Lyle et al., 1998, 1999). Using a genetic approach with the reporter mice with K15 – promoter-driven specific bulge hfSCs labeling confirmed that their descendent cells are able to build up all layers of the HF (Liu et al., 2003; Morris et al., 2004). While hfSCs are crucial to regenerate HF, bulge hfSCs can also contribute to wound repair but only temporarily and are not involved in long-term homeostasis of the epidermis (Ito et al., 2005; Levy et al., 2007).

Recently, it has been shown that the isthmus (a region of the HF directly above the bulge) is marked by Lgr6 (leucine-rich repeat-containing G protein-coupled receptor 6) (Figure 3A) and that these Lgr6+ cells assist in the homeostasis of the skin epidermis, sebaceous and HFs, they participate in skin epidermis regeneration after injury, and are long lasting (Joost et al., 2018; Snippert et al., 2010).

Above the isthmus in the HF, is a junctional zone containing the upper isthmus and lower infundibulum where the sebaceous gland opens into a hair follicle to secrete sebum. This structure also contains the SC populations which can reconstitute the epidermis, HF, and sebaceous gland in a reconstitution assay, and is labeled by MTS24 and Lrig1 (Leucine-rich repeats and immunoglobulin-like domain protein 1) expression (Figure 3A) (Nijhof et al., 2006; Jensen et al., 2008, 2009). Other research has shown that the junctional zone also hosts a different population of unipotent sebaceous gland progenitor cells labeled by Blimp1 (Figure 3A) (Horsley et al., 2006).

## Hair Germ Initiates Cyclic Hair Regeneration

It was originally believed that hfSCs occupied the secondary hair germ, at the bottom of the HF, during the quiescent phase of the HF cycle (Chase et al., 1951). Later research indicated that hfSCs in the bulge and HG cells are equivalently based on keratin15 expression as a specific marker to these cells (Lyle et al., 1998). Although hfSCs constitute the main source of cells in the growing phase of anagen, the HG is responsible for initiating a new hair cycle in direct reaction to molecular signals from the dermal signaling center (DP) at the beginning of anagen (Panteleyev et al., 2001; Greco et al., 2009). The HG is a transient region of the HF, that originated repeatedly from the hfSCs population at the resting phase of the hair cycle. In telogen phase HFs, the HG is located between the DP and bulge hfSCs (Figure 3A) (Ito et al., 2004; Greco et al., 2009; Zhang et al., 2009). The precise time point at which the HG starts to specify is still a topic of discussion. One hypothesis claims that the HG is formed directly from the hfSC population during the telogen phase, ignoring the fact that cells located below the bulge region can survive catagen (Greco et al., 2009; Zhang et al., 2009; Mesler et al., 2017; Panteleyev, 2018; Xin et al., 2018). A model reconciling both these hypotheses is proposed, in which such reconcilement is possible due to HG heterogeneity, visible through differential gene and protein expression in the telogen HG cells (Panteleyev, 2018). A different concept suggests that the HG originates from the hfSCs population present in the upper ORS (ORS-SCs). This characteristically located stem cell population is formed during the preceding anagen phase and after catagen survival and gives rise to the “new” unsymmetrical bulge with adjacent HG (Hsu et al., 2011). The HG is defined as being molecularly distinct from hfSCs by lacking hfSC markers such as NFATc1, S100A6, and CD34, yet it displays Lef-1 with specific expression of P-cadherin (Figure 3A) (Greco et al., 2009). Moreover, when comparing these two populations of cells at the transcriptional level, HG cells very closely resemble activated bulge hfSCs and function as a closely related, an extended, population of the bulge hfSCs (Ito et al., 2004; Greco et al., 2009). The hfSC and HG populations also express the same markers, such as SCs, K15, Lgr5, Sox9, Tcf3, and Lhx2 (Figure 3A) (Trempus et al., 2003; Greco et al., 2009). However, in contrast to hfSCs, isolated and cultured HG keratinocytes generate faster larger colonies but because of limited proliferative potential, they only grow effectively *in vitro* up to 3 passages (Greco et al., 2009). Interestingly, HG keratinocytes cannot maintain SC-like features *in vitro*. Current findings demonstrate that they possess the ability to functionally restore the bulge hfSCs population after injury, depilation, or laser ablation *in vivo*, indicating the reciprocal balance between both populations (Ito et al., 2004; Rompolas et al., 2012, 2013).

During telogen to anagen transition, HG cells exhibit faster activation than hfSC and HGs are a more dynamic population. They prepare for the onset of anagen by gradually increasing the expression level of genes involved in cell-cycle activation and signal transduction pathways (Greco et al., 2009). The first sign of HG proliferation is β-catenin stabilization along with other consequences of canonical WNT signaling activation. The network of genes involved in cell cycle progression is highly upregulated including a group of cyclins (Ccnb1, Ccna2, Ccnd2, and Ccnd1) and cyclin-dependent kinases (Cks2 and Cdc2a) (Greco et al., 2009). This highly correlates with the activation of WNT signaling in the ΔN-β-catenin mouse model with increased expression of the genes involved in cell cycle progression (Lowry et al., 2005). In addition, recent data demonstrates that at the onset of anagen, HG exchanging Tcf3/Tcf4 for Lef1 at the super-enhancers, and then Lef1 forms the nuclear complex with β-catenin after activation of WNT signaling (as a consequence of BMP inhibition) which drives HG committed progenitors (Merrill et al., 2001; Kobielak et al., 2007; Adam et al., 2018). The decreased BMP signaling observed in HG proliferation has also been promoted by activation of pSmad2 by transforming growth factor-β2 (TGFβ2) at the anagen onset (Oshimori and Fuchs, 2012). Recently, a two-step mechanism of early hair activation has been dissected further by showing the important role of DP in this process by restraining the bulge dependent inhibitors of WNT (such as Wif1, Frzb, Sost, Mest, Shisa3, and Igfbp4), which indirectly activate canonical WNT and Shh expression in HG progenitor cells to maintain a short period of proliferation in the lower part of hfSCs (Avigad Laron et al., 2018).

## Intrinsic Signaling Oscillation in hfSCs

The HF oscillation is the repetitive fluctuation between activation and quiescence phases of this mini-organ, leading to visible changes during regenerative hair cycles. Here, we explore how this biological fluctuation is orchestrated on a molecular level, leading to a periodic pattern of hair regeneration.

The recent discovery that BMP signaling maintains hfSCs in a dormant telogen, predominantly by inhibition of the canonical WNT signaling pathway, suggests a new model of intrinsic homeostasis regulation of these stem cells (Figure 3B) (Kandyba et al., 2013). Thus, any circumstances that reduce BMP signaling activity result in the upregulation of WNT canonical signaling with subsequent temporal hfSCs activation. Consequently, temporal stochastic activation of hfSCs tips the balance in favor of the dormant phase with high inner bulge BMP activity, thereby creating a cyclical molecular network (Kandyba et al., 2013; Li et al., 2015). Indeed, upon BMP inhibition, which leads to intrinsic hfSCs activation, Bmp6, and Gremlin1 (Grem1) are up- and down- regulated, respectively (Kandyba et al., 2013; Li et al., 2015). Endogenous Grem1 expression in feather buds is controlled by BMP signaling (Bardot et al., 2004), suggesting that activating and inhibiting components of this signaling axis are already reciprocally dependent and drive internal oscillation of gene networks in hfSCs (Figure 3B). In addition, a similar inverse relation was described between BMP signaling and the function of another inhibitor, Bambi, in hfSCs regulation (Kandyba et al., 2013). It was also reported that Bambi is antagonized by microRNA-31, which promotes a new hair regeneration cycle (Mardaryev et al., 2010), demonstrating the complexity of the main BMP signaling axis oscillation.

This intrinsic oscillation along the activator and inhibitor axis is consistent with the previous Turing model (Turing, 1990) which was expanded by Gierer and Meinhardt (1972). This concept describes a constant periodic pattern formation of opposing signals where one of the morphogens acts as an activator and the second one as an inhibitor, antagonizing the generation of the former to restrict the range of its activation spikes (Gierer and Meinhardt, 1972; Meinhardt and Gierer, 2000). The activator-inhibitor model proposes that the production of the agonist might promote the induction of its neutralizer – antagonist (Gierer and Meinhardt, 1972), which was also observed in hfSCs oscillation that stimulated opposed, yet synchronized, effects on the whole gene network (Kandyba et al., 2013).

**FIGURE 4:**
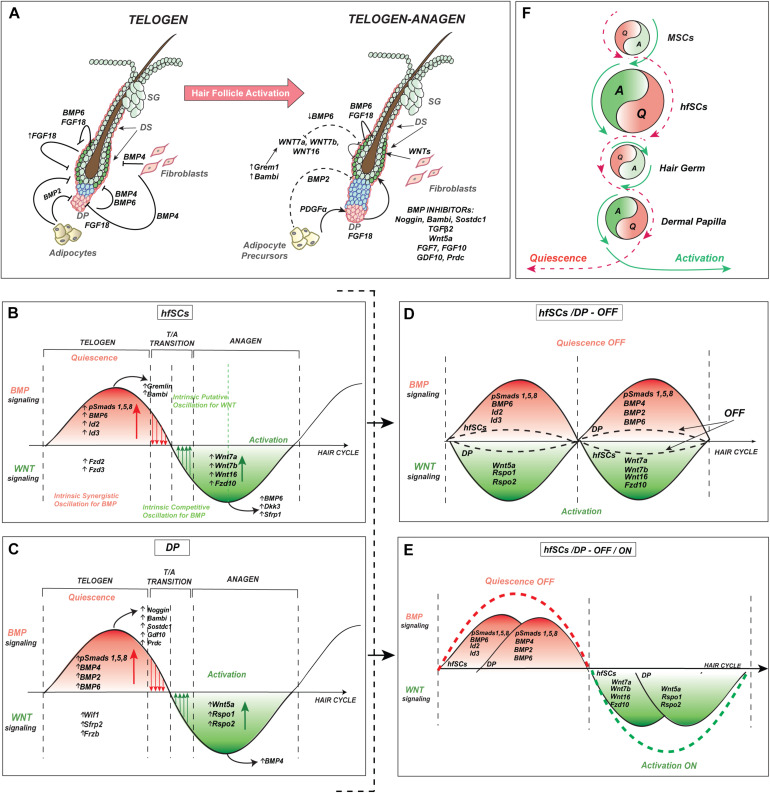
Intrinsic oscillator in hfSCs consolidates with extrinsic signaling in niche environment during hair cycle regeneration. **(A)** Proposed model of the molecular oscillation in hfSCs and its cross-talk during the quiescent-telogen and onset of HF activation during early anagen. The model also depicts intrinsic dynamic oscillation in hfSCs through observed rearrangements in ligand-receptor dependency and cross-talk between the main players of BMP and WNT signaling in SCs, and niche synchronized regulation. **(B)** Model of intrinsic oscillation in hfSCs between BMP and WNT signaling pathways, which sense and respond in an autocrine way to intra bulge hfSCs and in a paracrine way to the surrounding niche environment, including signaling center DP (intrinsic synergistic oscillation vs. intrinsic competitive oscillation with an accumulation of activators and/or inhibitors). **(C)** Model of internal feedback regulatory loop in DP between BMP and WNT signaling pathways, which sense and reciprocally respond to the hfSCs and the surrounding niche environment, especially bulge hfSCs in a paracrine way (synergistic vs. competitive accumulation of activators and/or inhibitors). **(D)** Model of wave interference in opposed phases between either hfSCs with repressive BMP and DP with stimulating WNT or in the reverse situation of hfSCs with activation of WNT and DP with inhibitory BMP signaling. The model of wave interference in opposed phases proposes attenuation with the inhibitory (OFF) effect of signals coming from either the intrinsic hfSCs oscillator or from the internal feedback regulatory loop of DP regardless of whether they are predominantly in BMP or WNT signaling. It results in reducing and flattening the wave amplitude, of BMP or WNT signaling either in hfSCs or DP (a dotted black sinusoid lines), creating a weaker resultant wave than individual waves before, and the mutual influence of these waves causes destructive interference. **(E)** Model of constructive interference with wave interference in the same stages (or in the same phases) of the repeating pattern waves between hfSCs and DP, with either repressive BMP resulting in quiescence (OFF) effect or stimulatory WNT leading to activation (ON) effect. The model of waves interference in the same phases proposes either inhibitory (OFF, a dotted red sinusoid line) effect or activatory (ON, a dotted green sinusoid line) effect of signals coming from either intrinsic hfSCs oscillator or internal feedback regulatory loop of DP and the phases depend on whether synchronization happens in BMP or WNT signaling for both hfSCs and DP. It creates a stronger resultant wave with a higher amplitude of either BMP (stronger inhibition) or WNT (stronger activation) signaling in both hfSCs and DP. **(F)** Model of the intrinsic oscillator in hfSCs which consolidates with extrinsic signaling in surrounding niche environment during hair cycle regeneration. MSCs, melanocyte stem cells.

This canonical BMP pathway suppression demonstrated that the intrinsic oscillation of the BMP signaling axis rearranges the WNT signaling pathway in a ligand-dependent fashion. As the result, the hfSCs molecular profile switched to mimic an HG destiny with canonical WNT activation (Kandyba et al., 2013). Consequently, BMP inhibition overcomes self-autonomous regulation with nuclear β-catenin stabilization in hfSCs, preceded by the upregulation of ligands: Wnt7a, Wnt7b, and Wnt16, followed by WNT inhibitor, Dkk3 downregulation (Figure 3B) (Kandyba et al., 2013). At the same time, hfSCs sense and respond to inhibition of BMP signaling resulting in a rearrangement of WNT receptors, called Frizzled (Fzd). In this arrangement, Fzd2 and Fzd3 must first be downregulated, simultaneously activating Fzd10, to prepare for canonical WNT activation (Figures 3B, 4B) (Kandyba et al., 2013). Recently, it has been confirmed that the regulatory element of the Fzd10 gene was closed during the quiescence of hfSCs and becomes more open and accessible, as demonstrated by the overlapping of ATAC-seq and Chip-seq data at the early stages of hfSCs activation (Adam et al., 2018).

Furthermore, a functional analysis confirmed that the subcutaneous introduction of the Wnt7a recombinant protein was capable of initiating a new cycle of HF regeneration with hfSCs activation (Ito et al., 2007; Kandyba et al., 2013; Kobielak et al., 2015). Conversely, when the Wnt7b gene was removed from hfSCs it postponed hfSC activation with a shorter anagen (Kandyba and Kobielak, 2014). The direct interaction of canonical BMP pathway effectors, phospho-Smads (pSmad1/5/8) has also been confirmed in the regulatory regions of Wnt7b and Wnt7a genes from *in vivo* isolated hfSCs (Kandyba et al., 2013; Genander et al., 2014; Kandyba and Kobielak, 2014). When the canonical Wnt pathway was inhibited by β-catenin ablation in hfSCs, it led to the upregulation of several BMP inhibitors including Grem1, Bambi, and Noggin, suggesting that WNT signaling suppresses BMP antagonist expression, permitting an increase in BMP signaling (Lim et al., 2016). This is consistent with the intrinsic oscillation model, which demonstrates that regulatory feedback loops are present between canonical BMP-WNT signaling axes and that a network of gene dependency maintains constant intrinsic oscillation in hfSCs (Figure 3B) (Kandyba et al., 2013; Lim et al., 2016).

Another interesting issue is the family of *Id* genes, which are highly sensitive to BMP stimulation. In hfSCs *Id1*, *Id2* and *Id3* are direct targets of pSmads in the canonical BMP signaling pathway, and are also significantly suppressed after *Bmpr1a* ablation (Kandyba et al., 2013; Genander et al., 2014). In other types of SCs *Id* genes are also involved in the preservation of cell identity and prevent premature differentiation (Niola et al., 2012).

This indicates that the regulatory feedback loops between BMP and WNT pathways sense either activation or inhibition of hfSCs during anagen or telogen. Moreover, they respond in opposite ways, either inhibiting hfSCs upon excessive SCs activation or activating hfSCs upon disproportionate SCs inhibition. This keeps the intrinsic oscillator constantly operating in stem cells, which introduces a check and balance mechanism for stem cell homeostasis (Figures 3B, 4B,C) (Kandyba et al., 2013).

## The Oscillation of hfSCs Is Under Antagonistic Influence of Inner Bulge Layer During Quiescence

The hypothesis in which multipotent and undifferentiated adult stem cells are seen in direct association with other cells within specific localization was first mentioned by Schofield (1978). This location represents a specialized microenvironment called a niche, which is maintained by the secretion of specific components of ECM to anchor in place and support the SCs. It is well established that niche cells arise from a different cell lineage than the SCs they regulate. Nonetheless, this concept is questioned as more recent niche studies in vertebrate and invertebrate systems consequently identify mechanisms where SCs progeny play a crucial niche element, providing and relaying signals that regulate SCs activity itself. This mode of action might be a substantial program controlling SCs behavior in homeostasis and wound repair. The HF inner bulge layer, positive for Keratin 6 (K6+), known as K6+ suprabasal bulge layer (SBL) is the inmost one, located between the telogen bulge hfSCs and the old HF called club hair (Figure 3A). It is widely believed that K6+ SBL cells arise from the hfSCs, exiting the bulge upon anagen onset, and then migrating and proliferating downward along with the exterior ORS. In the HF cycle progression, in late anagen, the ORS cells that survive the destructive phase of catagen atypically differentiate to form the inner bulge layer, SBL, in the newly established bulge region (Hsu et al., 2011). Although K6+ SBL maintains some of the hfSCs markers such as Lhx2, NFATc1, Sox9, Tcf3, and Foxc1 (Figure 3A) (Hsu et al., 2011; Lay et al., 2016; Wang et al., 2016), they are neither CD34 nor integrin positive. Regardless of SBL similarities to hfSCs, the former cannot replace SCs function in homeostasis or after an injury during wound repair. It seems that SBL’s predominant role is to maintain an attachment to the old hair shaft, therefore, preventing the animal’s hair loss when each hair cycle concludes. Interestingly, SBL extends to the K6 positive companion layer (Cp) separating ORS and IRS and both structures express anchoring proteins responsible for old hair club and old hair shaft hooking to the old bulge (Winter et al., 1998; Hanakawa et al., 2004; Lay et al., 2016; Wang et al., 2016). The high expression of Bmp6 and Fgf18 by SBL is sufficient to maintain hfSCs quiescence (Figure 4A) (Hsu et al., 2011) indicating the inner bulge layer’s crucial role in regulating hfSCs behavior. The removal of K6+ SBLs by hair plucking or genetic ablation leads to premature hfSCs activation and anagen initiation. Additionally, this precocious HF anagen can be blocked with subcutaneous injection of either Bmp6 or Fgf18, which is a primary example of how hfSCs progeny acts as a niche component maintaining hfSCS homeostasis *in vivo* – simply by restricting hfSCs activity through these signaling mechanisms (Blanpain et al., 2004; Greco et al., 2009). Although bulge cells express secreted WNT inhibitors such as Dickkopf 3 (Dkk3) and secreted frizzled-related protein 1 (Sfrp1) (Kandyba et al., 2013; Lim et al., 2016), usually associated with quiescent HF, the former one was found in the WNT-inactive internal bulge in the growth phase of the hair cycle (Lim et al., 2016).

## hfSCs Sense Surrounding Niche Components Forming Multifaceted Environments

In the case of hfSCs, the signals obtained from the niche determine their undifferentiated, multipotent state, and cycling status. The niche contains components of SCs progeny as well as heterologous cell types. In a niche centric view, the microenvironment provides signals necessary to maintain their stemness capacity. Recently, a mechanism indicating that SCs are born during embryogenesis at a stage that occurs long before the establishment of the HF bulge niche has been discovered (Ouspenskaia et al., 2016). The niche acts as an instructive signaling hub(s) that plays a critical role in maintaining SCs homeostasis and determines their behavior. Recent studies of hair follicle niche components have identified a number of additional layers in hfSCs regulation. For a better understanding of the reciprocal interactions during HF regeneration between hfSCs and a niche environment, this review will now discuss the key mechanisms that regulate the surrounding HF niche.

## Dermal Papilla and Dermal Sheath Integrate With the Intrinsic Oscillation in hfSCs and Hair Regeneration

At the end of the hair cycle, during telogen, HFs re-enter the quiescent phase and hfSCs remain dormant till the next anagen. It is generally assumed that during the telogen period, the activatory DP, attached to HG and indirectly to hfSCs, needs hefty inhibitory signals like Bmp4 from dermal fibroblasts and Bmp2 from adipocytes forming the neighboring macroenvironment to sustain hfSCs in the resting phase (Figure 4A). Toward the end of the telogen phase, the levels of repressive dermal BMP cues decline, resulting in increased expression of WNT proteins (Plikus et al., 2008; Plikus et al., 2011). In principle, crosstalk between the hfSCs and the neighboring niche at the local molecular level governs the switch from BMP-high/WNT-low inhibitory state to the BMP-low/WNT-high condition, which promotes the transition from telogen to anagen and a new hair regenerative cycle (Figure 4A). An essential structure that releases stimulatory factors to activate HFs is DP, a critical instructive signaling center that is part of the hfSCs niche attached to HG (Figure 4A). Recently reported lineage tracing studies have identified the existence of micro-niches alongside the DP axis. These newly discovered niches corresponded to the spatially arranged progenitors within the DP compartment, and are capable of differentiating into different epithelial layers within the hair follicle. These findings raised the possibility that by compartmentalization of progeny into distinct micro-niches where WNT-BMP crosstalk occurs, niches/tissues gain precise control over morphogenesis and regeneration (Yang et al., 2017). Several studies have shown, that during the postnatal HF cycle, DP regulates hfSC activity and promotes hair growth when grafted into HF, and coordinates with the epidermis in generating new HFs *in vivo* (Jahoda et al., 1984, 2001). However, DP cells without BMP receptor 1a (Bmpr1A) are not able to generate HFs in hair reconstitution assays (Rendl et al., 2008). A specific genetic approach with the development of transgenic mice, along with FACS technology, allowed for the isolation of a pure population of DP cells (Rendl et al., 2005). Still, the biggest challenge yet to be resolved, are problems related to the long-term maintenance of DP culture *in vitro*, because, after a few passages, DP cells lose their ability to induce HFs and usually become senescent (Horne et al., 1986). DP cells express several characteristic markers from which DP signature genes are selected. For example, secreted WNT inhibitors contain Wif1, Sfrp2, Frzb, and several BMP agonists like Bmp4 and Bmp6 along with BMP antagonists such as Noggin, Sostdc1/Ectodin/Wise, Gdf10, Prdc (protein related to Dan/Cerberus), and Bambi (Rendl et al., 2005) (Figure 4C). Importantly, changes in a gene expression pattern were dramatic only one passage after DP isolation (Rendl et al., 2005). These characteristic core genes indicate the complex signaling that controls DP properties, as well as HF inductive activity. One possible way of improving the culture method is to co-culture isolated DP cells with epidermal keratinocytes (Inamatsu et al., 1998) on extracellular matrix substrates (Havlickova et al., 2009) or with embryonic fibroblasts expressing Wnt3a or Wnt7a (Kishimoto et al., 2000). Other strategies to preserve the loss of trichogenicity assume the activation of the BMP signaling pathway (Rendl et al., 2008). Furthermore, the 3D spheroid culture of human DP cells preserved their *de novo* hair-inducing power for an extended time after combining with hfSCs in chamber graft regeneration (Higgins et al., 2013). In another, using 3D-printing technology, Abaci et al. (2018) generated a three-dimensional growth environment of human dermal papilla cells and successfully regenerated human HFs within human skin constructs. Apart from that, DP can repopulate the dermal sheath (DS) and the HFs surrounding dermal fibroblasts at the time of the reconstruction test and after injury (Rendl et al., 2008). Recent studies have characterized particular DP markers in the skin. Specifically, DP Sox2 expression was necessary for the formation of guard, awl, and auchene, but not zigzag hair (Driskell et al., 2009). Indeed, the genetic ablation of dermal Sox2 decreases the number of awl/auchene follicles with an increasing amount of zigzag hair, however, the overall number of HFs have not been affected during morphogenesis (all remained HFs stayed positive for another DP marker, CD133) (Driskell et al., 2009; Lesko et al., 2013). Moreover, Sox2 marks dermal cells sufficient to develop skin-derived precursors (SKPs) and these cell types are functionally similar and show similar gene expression profiles (Fernandes et al., 2004; Biernaskie et al., 2009). Endogenous and clonal, Sox2 positive, dermal cells functionally contribute to skin homeostasis by inducing hair morphogenesis, maintaining dermis, participating in wound-healing, and HF formation during transplantation, by functioning as dermal stem cells (Biernaskie et al., 2009). When HF is activated during the telogen-anagen transition, the change in DP gene expression causes the release of a plethora of stimulatory factors, such as Noggin and FGF pathway members, FGF7 and FGF10, collectively inducing hfSCs activation at the onset of anagen (Figure 4A) (Botchkarev et al., 1999; Kishimoto et al., 2000; Rendl et al., 2005, 2008; Driskell et al., 2009; Greco et al., 2009). A lack of β-catenin expression in DP in anagen HFs induced premature catagen and impaired hair regeneration, by downregulating FGF and IGF signaling pathways, unveiling coordinated reciprocal interaction between DP and epithelial progenitors (Enshell-Seijffers et al., 2010). Recently, the important role of DP in early anagen activation has been demonstrated by repressing the bulge-dependent inhibitors of WNT signaling, which induce canonical WNT and Shh expression in HG progenitor cells and indirectly promotes proliferation in the lower part of hfSCs (Avigad Laron et al., 2018). Furthermore, Rompolas et al. (2013) employed intravital imaging, combined with physical DP ablation to show that DP absence is sufficient to prevent anagen onset, therefore demonstrating DP significance during HF activation.

Recent studies that employed single-cell RNA sequencing (scRNA-seq) have defined a complete skin cell atlas comprising several different subpopulations, including HFs with the surrounding environment during the active and resting phases of the hair cycle (Joost et al., 2020). In the skin, seven fibroblast-like subpopulations have been recognized and classified as DP, DS, and skin fibroblasts. Four of those subpopulations experience prominent remodeling with expansion between the rest and growth phase of HFs (Joost et al., 2020). Furthermore, fibroblasts could be separated into four skin fibroblast subpopulations (FIB1–FIB4), DP into the anagen DP (aDP) and telogen DP (tDP), and DS appear to possess mature dermal sheath cells (DS2, predominantly observed in anagen), and one subtype with a transitional signature between dermal sheath cells (DS1) and skin fibroblasts (Joost et al., 2020). The results of single-molecule RNA-FISH demonstrated the existence of two DP subpopulations by staining for Notum, a deacetylase that inhibits WNT ligands specific for telogen DP cells, and Corin, specific for anagen DP cells (Enshell-Seijffers et al., 2008; Kakugawa et al., 2015; Joost et al., 2020). In terms of different expression markers, skin fibroblast subpopulations could be divided into fibroblasts representing the dermis (FIB1 and FIB2) and the hypodermis (FIB3 and FIB4) (Joost et al., 2020). This corresponds to previous studies in which lineage tracing experiments discovered two distinct lineages of skin fibroblast, representing the upper papillary dermis (CD26+, Blimp1+, Sca1− in mouse and CD26+, SFRP2+, FAP+, CD90− in human) and the lower reticular dermis (Dlk1+, in mouse and SFRP2+, FAP−, CD90+ in human), along with two pro-adipogenic types of fibroblasts (Sca1+, Dlk1−, and Sca1+, Dlk1+) (Driskell et al., 2013; Rinkevich et al., 2015; Salzer et al., 2018; Tabib et al., 2018; Korosec et al., 2019). In a transplantation assay, the papillary dermis forms DP and the arrector pili muscle, and reticular fibroblasts create ECM, preadipocytes, and adipocytes of the hypodermis (Driskell et al., 2013; Korosec et al., 2019). Evolutionary conservation in the papillary dermis between mouse and humans have been observed with the upregulation of components of the WNT signaling pathway such as Wif, Apcdd1, Rspo1, Axin2, Lef1, Wnt5a (Philippeos et al., 2018). During wound healing, the reticular and hypodermis fibroblasts respond first, then re-epithelialization must occur before papillary dermis regeneration (Driskell et al., 2013). Indeed, the upregulation of the canonical WNT signaling in the epidermis stimulates the expansion of the papillary dermis with HFs formation, as well as remodeling the lower dermis (Driskell et al., 2013). The upper dermis fibroblasts, labeled specifically by expression of CD26+, are responsible for scar formation during wound healing, and therefore, inhibition of CD26 significantly diminishes scaring in the skin (Rinkevich et al., 2015). However, as described by the lineage-tracing experiments during adulthood and then aging, the fibroblasts from the papillary layer contribute to the whole dermis, including mature adipocytes, and old fibroblasts increase WNT signaling (Salzer et al., 2018).

Regarding DS, recent discoveries have demonstrated that follicle-encapsulating DS cells are necessary for DP relocation to hfSCs/HG during catagen (Heitman et al., 2020). To facilitate this, DS cells must contract similarly to smooth muscle by activation of voltage-gated Ca^2+^ channels through calmodulin (CaM) → myosin light chain kinase (MLCK) → myosin heavy chain (MYH) and myosin light chain (MYL) with alpha-smooth muscle actin (αSMA) pathway allowing HFs regression movements (Heitman et al., 2020). Thus it seems that this machinery is crucial to maintaining a close proximity between hfSCs and DP cells (Heitman et al., 2020). Apart from the role to sustain mechanical proximity between hfSCs and DP during the catagen phase, DS cells also expressed genes like Fgf18 and Socs2 (Heitman et al., 2020). Thus, Fgf18 expression from outside of the bulge by DS adjacent cells adds an extra layer of regulation to previously described SBL to further integrate signaling and maintaining hfSCs quiescence (Hsu et al., 2011; Heitman et al., 2020). On the other hand, Socs2 is expressed in DS and DP and is known as a suppressor of cytokine signaling which negatively regulates the STAT5 signaling in DP for which inhibition has already been shown to delay the anagen phase, demonstrating yet another layer of signaling integration between different niche components influencing hfSCs quiescence (Udy et al., 1997; Legrand et al., 2016; Heitman et al., 2020).

All these discoveries about molecular DP characteristics suggest that DP might be under the reciprocal influence of hfSCs and other surrounding niche components (as discussed in the next part on subcutaneous adipocytes) to adequately sense and synchronously respond to either inhibiting or promoting HFs cyclic regeneration (Figures 4A–C). Since the main part of these molecular pathways mirror the changes observed in bulge hfSCs, regarding the intrinsic competitive cycling between BMP inhibitors and WNT activators and BMP activities in DP, it may suggest that this process is also intrinsic (Figures 4B,C). If this is a case, our working model considers the presence of at least one intrinsic oscillator localized in hfSCs and might predict the existence of a second putative one in DPs during hair regeneration (Figures 4B,C,F).

## Subcutaneous Fat and Its Precursors Interplay With the Intrinsic hfSCs Oscillator

The behavior of hfSCs is controlled by the regulatory factors provided by DS fibroblasts adjacent to the HG and the hfSCs (Figure 4A). Additionally, it was shown that dermal white adipose tissue (DWAT), an important regulator of hfSC activity, is also in close contact with hfSCs (Zwick et al., 2018). Waves of inhibitory and activating signals, produced by the subcutaneous fat cells, facilitate the synchronization of hfSCs, primarily affecting DS and DP activity (Figure 4A). It has been revealed that during the hair cycle, the thickness of DWAT shows parallel and synchronized oscillation to HFs, as they are thicker in anagen and thinner in telogen (Chase et al., 1953; Hansen et al., 1984; Festa et al., 2011; Nicu et al., 2019). The enlargement of DWAT during the early anagen is caused by the proliferation and differentiation of preadipocytes, as well as the hypertrophy of adipocytes (Rodeheffer et al., 2008; Festa et al., 2011). Adipogenesis is regulated by WNT/β-catenin and Shh signaling from the growing HFs (Donati et al., 2014; Zhang et al., 2016). In contrast, during the anagen-to-catagen transition, DWAT shrinks, which is caused by lipolytic change and the dedifferentiation of mature adipocytes (Nicu et al., 2019; Zhang et al., 2019). Notably, during the telogen-to-anagen transition, the amount of DWAT increases (Chase et al., 1953; Plikus et al., 2008; Festa et al., 2011). Subcutaneous adipocytes periodically express Bmp2 and Bmp4 to inhibit hfSCs activity and maintain the quiescence and the refractory telogen state (Plikus et al., 2008). The activity of hfSCs can also be suppressed by other dermal inhibitors, such as secreted frizzled-related protein 4 (SFRP-4) and DKK1 (Plikus et al., 2011). DWAT also produces activators including follistatin, which leads to stimulation of hair wave propagation during anagen (Chen et al., 2014). Intradermal immature adipocytes were shown to play a crucial role in controlling the hfSCs through platelet-derived growth factor (PGDF) signaling which stimulates DP cells to activate hair cyclic regeneration (Figure 4A) (Festa et al., 2011).

There are some examples of genetically modified mouse models, including mice with human fatty acid transport protein (FATP4) and apolipoprotein C-I overexpression, or mice deficient for Dgat1 and Dgat2, all developing changes in the adipocyte layer, HFs, and skin structure (Jong et al., 1998; Chen et al., 2002; Herrmann et al., 2003). Other research demonstrated a surprising discovery that fat cells can be regenerated from myofibroblasts during wound healing. For cell lineage reprogramming, neogenic HFs around a wound are necessary to trigger BMP signaling to activate adipocyte-specific transcription factors (Plikus et al., 2017).

Reciprocal signaling and interaction between HFs and DWAT demonstrate the importance of the relationship between hfSCs and their niche to sustain proper skin homeostasis. Although a great deal remains unclear about the comprehensive crosstalk between the HF niche cells, during the various stages of the HF cycle the behavior of hfSCs are largely controlled by the different niche components (Plikus et al., 2008, 2011; Festa et al., 2011).

## Extra Players Influencing Intrinsic hfSCs Oscillation and Hair Regeneration

Although still a lot of things remain undiscovered about the comprehensive crosstalk between the HF niche cells, more compound perspectives are appearing in research, showing that during the various stages of the HF cycle the behavior of hfSCs is controlled by the different niche components (Jahoda et al., 1984; Botchkarev et al., 1999; Rendl et al., 2005; Plikus et al., 2008; Festa et al., 2011; Hsu et al., 2011; Kobielak et al., 2015; Joost et al., 2020).

Four types of sensory neurons are concentrated on the top bulge region of the HF to ensure that the sensory responses coordinate the behavior of SCs within the skin (Abraira and Ginty, 2013; Li and Ginty, 2014). Recently it has been found that sensory nerves release Shh to a pool of perineurally located Shh-sensitive upper bulge SCs (Gli1 positive) that permanently participate in wound healing. Hence, their lineage can change into the epidermal SCs, demonstrating the crucial role of the Shh signal to transform and sustain the upper bulge keratinocytes into an epidermal fate (Figure 3A) (Brownell et al., 2011). During development, Shh signaling also plays a critical role in the arrector pili muscle-sympathetic nerve niche formation, which in light of recent studies, regulates hair follicle stem cell activity. In this dual-component niche system, norepinephrine, a neurotransmitter, is secreted by sympathetic nerves which activate hfSCs and promote anagen entry. Additionally, the loss of norepinephrine signaling causes stem cell quiescence by down-regulation of cell-cycle and metabolic machinery, as well as up-regulation of two quiescence regulators Fgf18 and Foxp1 (Shwartz et al., 2020).

The HF bulge area is surrounded by blood vessels (part of dermal vasculature – superficial vascular plexus) that provide the nutritional and hormonal input into the hfSCs population, which was confirmed by recent research illustrating the connection between the bulge SCs and the perivascular niche (Amoh et al., 2004; Xiao et al., 2013). Additional research has confirmed that the hair cycle depends on cutaneous angiogenesis and identified Bmp4 regulation as one of the main inhibitory factors of hfSCs anagen progression (Mecklenburg et al., 2000; Li et al., 2019). Furthermore, the K15 negative upper bulge cells located adjacent to the vascular annulus express a signaling factor EGF-like domain- multiple 6 (Egfl6), which is involved in angiogenesis and the recruitment of vascular endothelial cells (Xiao et al., 2013).

A recent study also highlighted that lymphatic remodeling, driven by hfSCs, coordinates HFs regeneration by controlling the composition of fluids and cells in the surrounding environment. HfSCs control the behavior of lymphatic capillaries through the secretion of molecules Angptl7 and Angptl4, which act as an on/off switch for drainage. The niche connection between lymphatic and hfSCs revealed its importance in maintaining the balance between hfSCs self-renewal and quiescence. This novel concept represents a potential source of new therapeutic targets for lymph-related conditions, including hair loss and wound-healing (Gur-Cohen et al., 2019; Pena-Jimenez et al., 2019).

Hair pigmentation depends on the production of melanocytes and their melanin-product deposition inside keratinocytes. Melanocytes come from melanocyte stem cells (MSCs) during each hair cycle activation. During development, MSCs derive from the neural crest and are located in the bulge area, interspersed between hfSCs (Nishimura et al., 2002). The coordinated mutual interaction between MSCs and hfSCs allows for sMSCs activation and differentiation into melanocytes which produce and transfer melanin to the bulb matrix progenitors during each hair regeneration to produce hair color (Tanimura et al., 2011). The coordinated activation of canonical WNT signaling and collaboration between hfSCs and MSCs especially through Wnt7a and Wnt10b ligand, the first, previously described in the intrinsic oscillation model, initiates pigmented hair regeneration. Thus, it suggests that this crosstalk between SCs in the bulge is necessary to couple their behavior and coordinate the outcome of hair regeneration and pigmentation (Figure 4F) (Rabbani et al., 2011; Chang et al., 2013). The skin pigmentation pattern named “anagen-coupled melanogenesis” can also serve as a sign of the hfSCs activation, which is manifested by visible pigment production during each new hair cycle (Chase and Eaton, 1959).

Immune cells are crucial for proper hair development, not only during embryogenesis but also to regulate the regeneration of HFs during physiological hair cycling and after skin injury by stimulating the proliferation and differentiation of hfSCs (Paus et al., 1998; Gay et al., 2013; Ali et al., 2017; Lee et al., 2017; Wang et al., 2017; Rahmani et al., 2018). Although it has been shown that T cells are associated with the hair follicle throughout the entire hair growth cycle, they are most abundant during the rest phase, as they participate in telogen to anagen transition. Under normal physiological conditions, regulatory T cells (Tregs), which express skin-resident Foxp3 (forkhead box p3), are located in the follicular epithelium close to hfSCs. They evoke differentiation and proliferation of hfSCs through the Jag1-Notch pathway (Zhang et al., 2019). Research showed that activation of γδ T cells promotes hair growth and helps the wound healing process by stimulation of interfollicular epidermal basal cells (Jameson and Havran, 2007; Lee et al., 2017). The γδ T cells are necessary for HFs neogenesis by secretion Fgf9 that subsequently activate WNT in fibroblasts in the wounded area (Gay et al., 2013). However, immune privilege collapsing may lead to an immune attack on HFs, resulting in diseases such as Alopecia areata, immunity-mediated hair loss (Paus et al., 2003; Gilhar et al., 2007; Strazzulla et al., 2018). Although the exact cause of this disease remains unknown, most probably it occurs due to increased presentation of autoantigens in surveying T cells which can recognize HF antigen resulting in autoimmune responses against HFs (Gilhar et al., 2007; Strazzulla et al., 2018). The described inflammatory response mainly attacks the lower transient segment of anagen HFs, inhibiting hair growth, and promoting premature anagen-to-telogen transition (Strazzulla et al., 2018). In summary, the influx of autoreacting T cells into the HFSC niche is one of the elements that prevent normal hair growth.

The other immune cells that are located in the HF niche and that have been shown to regulate the hair cycle are macrophages (Paus et al., 1998; Castellana et al., 2014). Macrophages in the skin exist as a heterogeneous population, therefore they can have different effects on hfSCs (Castellana et al., 2014). They occur most abundantly during the telogen state (Castellana et al., 2014). Reduction in the number of macrophages initiates premature anagen onset, while apoptotic macrophages can activate hfSCs via WNT signaling, releasing Wnt7b and Wnt10a ligands (Castellana et al., 2014). In contrast, other studies on murine macrophages during telogen, which utilized scRNA-seq, led to the discovery of TREM2+ dermal macrophages, which can secrete oncostatin M (OSM), a possible antagonist of hfSCs cyclic regeneration (Yu et al., 2008; Wang et al., 2019). OSM activates the JAK-STAT5 signaling pathway, maintaining the hfSCs in the quiescence state during the telogen state (Wang et al., 2019). Therefore, the ablation of these particular macrophages leads to an anagen state (Wang et al., 2019). Additionally, macrophages also play a role in the hfSCs response to injury. Hitherto, several ways of activating macrophages that are caused by wounds to the skin have been already described (Ito et al., 2002; Osaka et al., 2007; Chen et al., 2015). In particular, CX3CR1 high CCR2+ macrophages expressing TGF-β1 and TNF-α play a crucial role in hair growth promoted by injury (Rahmani et al., 2018). Macrophages secreting TNF-α lead to hfSCs activation through the TNF-α/p-AKT/p-β-catenin-Ser552 signaling axis, resulting in anagen onset in the skin of surrounding wounds (Osaka et al., 2007; Wu et al., 2015; Rahmani et al., 2018). Taking it all together, macrophages are potential candidates in promoting hair growth. The activation of WNT/β-catenin signaling can promote their proliferation and accumulation, therefore participating in hair and skin regeneration.

## The Interplay Between the Extrinsic Niche Environment and the Intrinsic hfSCs Oscillator During Homeostasis and Hair Cycle Regeneration

Stem cell reservoirs are localized in dedicated niches that serve as *de facto* macro- and micro- environments that control SCs homeostasis regulation (Spradling et al., 2001; Lin, 2002; Fuchs et al., 2004). HfSCs also release self-stimulating agonists in a self-autonomous way at the initiation of growth while maintaining dormancy throughout the majority of the adult HFs cycle, however, the underlying mechanisms of this process are not well understood (Blanpain et al., 2004; Greco et al., 2009; Zhang et al., 2009; Oshimori and Fuchs, 2012).

Bone morphogenetic protein signaling is one of the most important pathways necessary for correct HFs cyclic regeneration (Botchkarev et al., 2001; Kobielak et al., 2003, 2007; Andl et al., 2004; Plikus et al., 2004; Zhang et al., 2006). HFs ORS, along with bulge hfSCs, exclusively express transmembrane Bmpr1a which is essential to transduce canonical BMP signal after binding with secreted BMP ligands (Bmp2/4/6) by the phosphorylation of Smad transcription factors (pSmad1/5/8) (Andl et al., 2004; Kobielak et al., 2007). After activation of BMP signaling, pSmad1/5/8 forms a heterodimer with Smad4 to *trans*-activate specific target genes in the nucleus during quiescent telogen (Andl et al., 2004; Massague et al., 2005; Kobielak et al., 2007). The genetic removal of the *Bmpr1a* in the HFs contributes to premature and prolonged stimulation of dormant hfSCs with perturbed homeostasis (Kobielak et al., 2007), which results in specific hair tumor formation (Kobielak et al., 2003, 2007; Andl et al., 2004; Zhang et al., 2006). BMP agonists or antagonists are secreted by either intrinsic hfSCs oscillator or by external niche during HFs cyclic regeneration. During the onset of a new anagen, opposing changes in expression occur with down-regulated Bmp4 and up-regulated Noggin, which is simultaneously observed in an extrinsic niche (DP) and intrinsic HG (Figures 4A–D) (Botchkarev et al., 2001). Bmpr1a is also expressed in the DP transducing canonical BMP pathway to form HFs (Rendl et al., 2008) and BMP activity fluctuation in the DP results in the generation of distinctive HFs set (Driskell et al., 2009).

Despite the prevailing and crucial function of DP in niche stimulation or suppression of hfSCs, the subcutaneous fat adjacent to hfSCs and HG was shown to release Bmp2 and Bmp4, thus demonstrating that it is an important player in the hfSCs environment (Plikus et al., 2008; Festa et al., 2011). In addition, intrinsic up-regulation of Bmp6 has been proposed to suppress hfSCs, keeping them in a dormant state (Figure 4A) (Blanpain et al., 2004; Kandyba et al., 2013).

Bone morphogenetic protein signaling play a fundamental role in SCs homeostasis in several organs in the body (Zhang et al., 2003; Haramis et al., 2004; He et al., 2004; Kobielak et al., 2007). Interestingly, the crucial role of BmpR1A has also been confirmed in hematopoietic stem cells (HSCs), in the intestinal stem cells (ISCs), and more recently in neural stem cells (NSCs) to maintain quiescence of adult SCs by activation of canonical BMP signaling (Zhang et al., 2003; Haramis et al., 2004; He et al., 2004; Mira et al., 2010). In contrast, in embryonic SCs (ESCs) BMP ligands work together with LIF to preserve stemness and promote proliferative conditions in cell culture (Sato et al., 2003; Ying et al., 2003). Similarly, an invertebrate homolog of BMP ligand, *dpp* is needed to set a niche to preserve anterior germline SCs in the fly embryo (Xie and Spradling, 1998).

On the other hand, the WNT signaling with β-catenin nuclear accumulation had been shown as the first pathway crucial to stimulating HFs anagen by promoting dormant hfSCs to proliferate (Gat et al., 1998; Van Mater et al., 2003; Lo Celso et al., 2004; Lowry et al., 2005). Indeed, the overexpression of the stabilized nuclear form of β-catenin in the HFs of genetically modified mouse models resulted in early hfSCs stimulation, which later led to the development of HFs derived tumors, pilomatricomas, and trichofolliculomas (Gat et al., 1998; DasGupta and Fuchs, 1999). A similar genetic mutation that stabilized β-catenin was found in approximately three-quarters of human pilomatricomas (Chan et al., 1999). In contrast, when β-catenin was genetically removed from hfSCs it caused hfSCs to lose their ability to stimulate new HFs growth, and to maintain the fate of HF progenies, preferentially adapting IFE characteristics (Huelsken et al., 2001; Lowry et al., 2005). After the inside interaction between BMP signaling and the WNT pathway was described in ISCs, where suppression of BMP leads to PTEN inhibition with subsequent β-catenin stabilization (He et al., 2004), a similar probable link between both pathways was suggested in hfSCs (Kobielak et al., 2007). HFs tumors resembling trichofolliculomas were formed after Noggin overexpression that neutralized the BMP pathway, thus linking it directly to WNT signaling (Sharov et al., 2009).

One of the well-defined processes of hfSCs stimulation is the upregulation of WNT pathway after the suppression of the BMP signaling pathway, with nuclear β-catenin stabilization at first in HG, then in hfSCs to push HFs cyclic regeneration (Gat et al., 1998; Botchkarev et al., 1999; Kobielak et al., 2003, 2007; Van Mater et al., 2003; Andl et al., 2004; Lo Celso et al., 2004; Zhang et al., 2006; Ito et al., 2007; Plikus et al., 2008; Greco et al., 2009; Plikus et al., 2011). Until recently, there was a missing link in understanding of how the BMP pathway controls canonical WNT signaling on the molecular level to influence a complex gene network that promotes hfSCs stimulation. A novel machinery that internally integrates a gene network between BMP and WNT signaling was recently uncovered precisely in hfSCs, where the suppression of the BMP pathway boosted the intra-bulge WNT ligands (Figures 3B, 4A) (Kandyba et al., 2013). HGs and hfSCs cells reach early anagen activation after the DP release BMP antagonists, such as Noggin (Figures 4A,C) (Botchkarev et al., 1999; Botchkarev et al., 2001; Greco et al., 2009). The study assumed that the hfSCs were randomly activated in the next hair cycle and that they must be in the right oscillation phase to respond to DP secreted Noggin by the preceding intrinsic secretion of Grem1 and Bambi by the hfSCs oscillator during the high BMP refractory phase of telogen (Figure 4B) (Kandyba et al., 2013). Thus, BMP inhibition from external and internal sources must further prepare stochastically selected hfSCs to respond to anagen activation by upregulation of the canonical WNT receptor, Fzd10 with simultaneous downregulation Fzd2 and Fzd3 (non-canonical ones) (Figure 4B) (Kandyba et al., 2013; Adam et al., 2018). At the same time, there is observed overexpression of the intra-bulge WNT ligands like Wnt7a, Wnt7b, and Wnt16 after inhibition of BMP/pSmads1/5/8 signaling (Figures 3B, 4A) (Kandyba et al., 2013; Genander et al., 2014; Kandyba and Kobielak, 2014). Functionally, the biological consequences of WNT canonical ligand upregulation were confirmed by the subcutaneous introduction of the recombinant protein of Wnt7a, which resulted in hfSCs stimulation, initiating a new regeneration cycle (Kandyba et al., 2013). Collectively, these findings on internal antagonistic competition underline the existence of a previously unknown intrinsic oscillation between the signaling of BMP and WNT gene networks within hfSCs. This fluctuation counterbalances SCs action indicating that halted BMP releases the inhibition of WNT, which forces individual hfSC to the anagen phase, proportionally to the signal strength changes between BMP/WNT (Figures 3B, 4A). These findings propose an additional chain of command to control hfSCs besides the reciprocal interaction between bulge and DP, as well as HFs and subcutaneous fat (Kandyba et al., 2013). Thus, canonical BMP and WNT pathways might serve as one of the common signaling platforms for the reciprocal communication between some extrinsic niche components and hfSCs, allowing SCs to appropriately analyze the surrounding signals to adequately respond during new hair cycles (Kandyba et al., 2013).

Dermal papilla and Noggin secrete transforming growth factor-β2 (TGFβ2) simultaneously during anagen onset (Figure 4A) (Oshimori and Fuchs, 2012). TGFβ2 works synergistically with Noggin and causes HG to respond with the upregulation of TMEFF1 (transmembrane protein with EGF-like and two follistatin-like domains 1), which further suppresses BMP signaling (Figure 4A) (Oshimori and Fuchs, 2012). Additionally, PDGFα (platelet-derived growth factor-α) is produced by precursors of intradermal fat and activates the dermal cells adjacent to hfSCs, promoting anagen (Figure 4A) (Festa et al., 2011). Thus, hfSCs sense the number of surrounding niche cues and respond accordingly to them with activation of HFs anagen, if the niche cues for activation are more prevalent than inhibitory ones (Figure 4A) (Oshimori and Fuchs, 2012).

The exact molecular mechanism of the WNT pathway in the activation and proliferation of hfSCs is still unveiling, but recent studies shed light on these intricate interactions. Based on microarray and ChIP analyses, Cyclin D1 was identified as one of the up-regulated cell cycle-promoting factors, upon nuclear β-catenin stabilization, during the beginning of telogen, when SCs become activated and start to proliferate (Lowry et al., 2005). This finding is consistent with previously established chromatin immunoprecipitation (ChIP) assays, where Cyclin D1 was identified as one of the WNT target genes (Chamorro et al., 2005). Moreover, chromatin remodeling factors: Timp3, and Hmgn3, containing Lef1/Tcf-binding sites, were identified as direct genes activated by the β-catenin chromatin complex during telogen- anagen transition (Lowry et al., 2005). Indeed, it seems that switching Tcf3/Tcf4 for Lef1 and nuclear complex Lef1/β-catenin formation after activation of WNT signaling is crucial for anagen onset (Adam et al., 2018).

The length of HF cycle phases is appropriately regulated by DP and the subcutaneous fat adjacent to hfSCs and the SBL of HFs, which cooperatively generate signal modulating hubs by balancing the molecular gene network in SCs (Figure 4A) (Plikus et al., 2008, 2011; Greco et al., 2009; Festa et al., 2011; Hsu et al., 2011). Furthermore, the performance of an individual hfSC is adjusted randomly by periodic and recurring interplays. These may have various consequences that depend on changes in the stimulatory or dormant landscape, influenced by the general macro- or more regional local micro- environment (Figure 4F) (Chuong and Widelitz, 2009). There are also two phases of telogen that are marked by the differential expression of dermal BMP, known as refractory and competent telogen according to high or low levels of BMP, which are in opposite phases to canonical WNT signaling (Plikus et al., 2008).

These discoveries have established a novel reciprocal dependency where canonical BMP signaling trumps the canonical WNT pathway in terms of the molecular control of the intrinsic oscillation of hfSCs homeostasis during dormancy and activation (Figures 3B, 4A,B), further integrating the signaling of the surrounding extrinsic niche environment. This model of the intrinsic oscillator within hfSCs might explain how different levels of internal and external signals merge, in either compatible or incompatible phases. This results in either hastened or deferred hfSCs receptiveness to activation by shortening or lengthening the refractory and competent telogen (Kandyba et al., 2013, 2014; Kandyba and Kobielak, 2014; Kobielak et al., 2015). The presence of the hfSCs oscillator, which is responsive to either their own repressive BMP signals or to those of the extrinsic niche environment, resembles the oscillating states described in pattern formation during morphogenesis (Maini et al., 2006). Thus, the permanent intra-bulge and mutually exclusive oscillation between agonists and antagonists are essential for the preservation of hfSCs homeostasis.

Since the activation with WNT or inhibition with BMP and the fact that signaling could be represented as respective waves (Figures 4B,C), we propose that the intrinsic oscillator in hfSCs (or the second putative independent one in DP) has two phases, where each of them follows after the other in a constantly alternating rhythm, with the canonical BMP pathway upstream of WNT signaling (Figure 4B, intrinsic oscillation for BMP either in synergistic or competitive phase) (Kandyba et al., 2013). It is important to note that hfSCs react to the phase of this rhythm by sensing the level of active pSmads that represent inhibitory canonical BMP signaling, causing either repression of the WNT pathway (BMP/pSmads high) or activation of WNT (BMP/pSmads low) (Figures 4B,C). Despite the BMP axis, the WNT pathway itself is a second axis that maintains this constant rhythm, where the canonical nuclear stabilization of β-catenin forces the activation of Bmp6 as well as their inhibitor Dkk3 and Sfrp1, thus allowing WNT to reactivate Bmp with inhibition of activated hfSCs (Figure 4B, putative intrinsic oscillation for WNT) (Kandyba et al., 2013; Lim et al., 2016). By these means, stem cells possess an intrinsic regulatory network mechanism that works contrary to the phase of either activation or inhibition, thus maintaining balance and preventing either overactivation (with tumor formation) or over inhibition (with suppression of stem cell homeostasis or stemness).

Taking these findings into account, we, therefore, propose that the intrinsic oscillator in hfSCs may interact with the extrinsic surrounding niche environment. To simplify our model, we performed a comparison with the DP niche, where cells from these different compartments may meet with either opposite waves of WNT and BMP signaling, or in the same waves of these pathways (just for extreme wave interference situations). In the first model, if the opposing waves of WNT and BMP signaling interfere in reverse phases, the prediction will be to observe the attenuation of emerging signals coming from the intrinsic hfSCs oscillator and DP with inhibitory effects (Figure 4D, OFF). Consequently, it will reduce the wave amplitude of BMP or WNT signaling either in hfSCs or DP and creating a weaker (than each of waves that merged before) and more flat wave, leading to destructive interference (Figure 4D, a dotted black sinusoid lines, OFF). On the contrary, the model of wave interference in concordant phases between hfSCs and DP proposes either an inhibitory effect when waves synchronize in the same stage of BMP signaling (Figure 4E, OFF, a dotted red sinusoid line) or an activating effect if waves merge in the same stage of WNT signaling (Figure 4E, ON, a dotted green sinusoid line). This results in a stronger wave with a higher amplitude of either BMP (stronger inhibition) or WNT (stronger activation) signaling for both hfSCs and DP, causing a situation of constructive interference (Figure 4E, OFF and ON).

In the future, these models might be useful in building a computational design that predicts HFs behavior in terms of mutual interaction with the external niche environment during the hair cycle.

## Discussion

This model proposes that the intrinsic oscillation of signals in hfSCs is critical in forming and maintaining a periodic pattern in the cycle of hair regeneration. If stem cells and the surrounding environment niche had a predisposition to reach a constant balance, there would be no spontaneous provoking cues that initiate another hair regeneration cycle. Therefore, the presented model assumes a need for a dynamic oscillating gene network in the stem cell population instead of a static one, which rhythmically fluctuates. It reaches amplitude in any of the two divergent hfSCs states by sensing and responding to surrounding signals (Figures 4B,C). As a result, the oscillation of the intrinsic signals is crucial for the stochastic initiation of spontaneous hair cycle activation or its quiescence (Figure 3B). Therefore, the regenerative system of HFs has at least one stem cell population intrinsically oscillating and constantly tipping the balance in the favor of either quiescence or activation. Simultaneously, this mini-organ maintains a dynamic self-responding gene network that is primed to be stimulated in an active state or silenced in a quiescent one (Figure 3B).

Furthermore, the BMP axis might be one of the key components of the signaling pathway, orchestrating intrinsic stem cell oscillation in HF in an *in vivo* model (Kandyba et al., 2013; Genander et al., 2014). Therefore, this model hypothesizes that the BMP pathway is upstream of the others and defines a center axis of the intrinsic hfSCs oscillation. To date, other well-documented studies have confirmed the existence of canonical BMP-WNT signaling axes that behave like an intrinsic oscillator, resulting in an antagonistic competition between BMP and WNT gene networks in hfSCs (Figures 3B, 4A) (Kandyba et al., 2013; Genander et al., 2014; Adam et al., 2018). These studies were recently supported by the discovery of antagonistic competition between WNT and BMP gene networks when the canonical WNT pathway was inhibited by β-catenin ablation in hfSCs, adding a new gene network dependency to maintain constant intrinsic oscillation in hfSCs (Figure 3B) (Kandyba et al., 2013; Lim et al., 2016). Furthermore, this indicates that the regulatory feedback loops between canonical BMP and WNT pathways not only sense the change of hfSCs activity but that they can respond accordingly to regulate hfSCs. This pathway interaction keeps the intrinsic oscillator of the stem cells constantly active, introducing a check and balance mechanism to maintain stem cells homeostasis (Figures 3B, 4B,D) (Kandyba et al., 2013; Genander et al., 2014; Lim et al., 2016; Adam et al., 2018).

As presented in this review, data suggest that the cyclic regeneration of hair might depend at least on one intrinsic oscillator located in hfSCs that maintains the regeneration rhythm of HF. It would, therefore, be intriguing for future studies to explore whether other components of the hair mini-organ discussed here, such as DP (the second putative independent intrinsic oscillator), also possess such essential oscillation, or whether they just respond to intrinsic clues from the oscillation in hfSCs. It could be presumed that some other stem cell-based regenerative systems might possess a similar system of regulation. Therefore, it would be interesting to investigate if they could maintain their rhythm, by either initiating a subsequent cycle of regeneration or maintaining quiescence.

In conclusion, the model of hfSCs is very instructive for research on how adult SCs, their descendants, and the extrinsic niche environment equalize or cooperate to sustain SCs responsiveness and reactivity during homeostasis. Within the skin, a highly compartmentalized niche forms the macroenvironment in which elements are tightly connected with each other and depend on adipocytes or their precursors, the immune system with macrophages and cells composed directly HF, for example, DP or the K6^+^ Inner Layer.

Although hfSCs release controlling factors in a self-autonomous way, they stochastically respond to external niche signals and manage mini-organ regenerative urgency, which might be a universal SCs controlling mechanism. Ultimately, the discovery of the mutual interaction and gene networks among different extrinsic niche elements and hfSCs will contribute very useful information that might be applicable in stem cell-based medical therapies. Further understanding of the composition and the signals within the oscillator and environment of the hair mini-organ might speed up the development of potent small-molecule based therapy to safely initiate the regenerative cycle of the hair follicle.

## Author Contributions

KK contributed to the conception and design of the manuscript. PD, PM, TDP, AO, ŁMB, and KK wrote the article and approved the final version. All authors read and approved the final manuscript.

## Conflict of Interest

The authors declare that the research was conducted in the absence of any commercial or financial relationships that could be construed as a potential conflict of interest.
